# Supplementary figures and images for: P Starvation in Roses Leads to Strongly Genotype-Dependent Induction of P-Transporter Genes during Black Spot Leaf Disease
Source: J Fungi (Basel). 2022 May 24;8(6):549. doi: 10.3390/jof8060549 (PMC9224717; doi:10.3390/jof8060549)

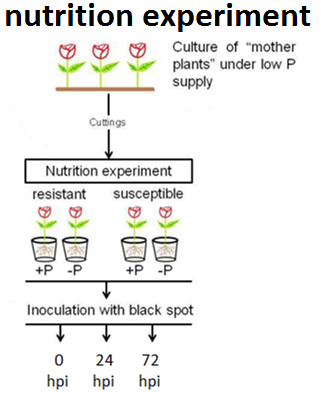

Supplement: Supplementary file 1 [file jof-08-00549-s001.zip › FigureS1.tiff]

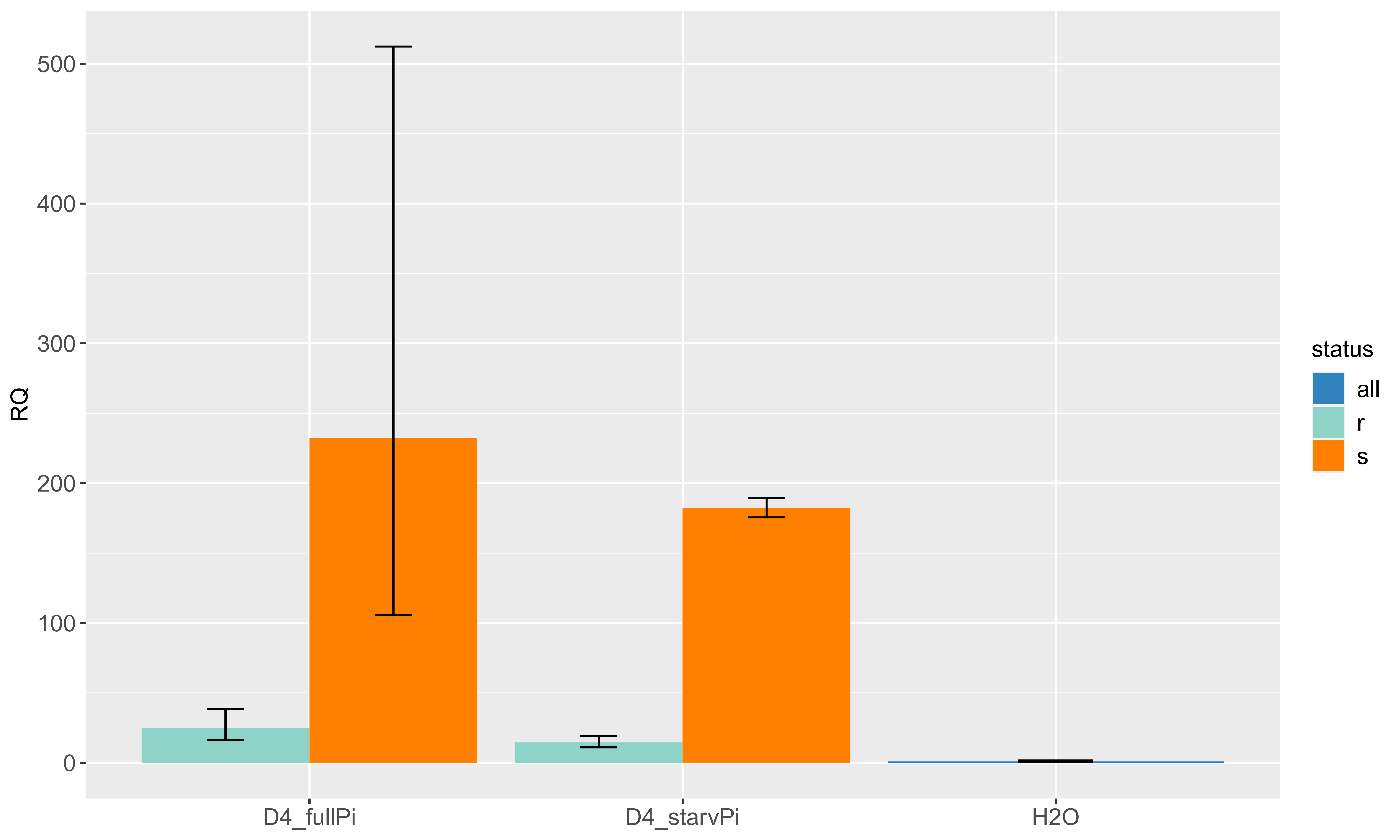

Supplement: Supplementary file 1 [file jof-08-00549-s001.zip › FigureS2.tiff]
